# Heterozygous deletion of *SCN2A* and *SCN3A* in a patient with autism spectrum disorder and Tourette syndrome: a case report

**DOI:** 10.1186/s12888-018-1822-8

**Published:** 2018-08-02

**Authors:** Kathrin Nickel, Ludger Tebartz van Elst, Katharina Domschke, Birgitta Gläser, Friedrich Stock, Dominique Endres, Simon Maier, Andreas Riedel

**Affiliations:** 1 Section for Experimental Neuropsychiatry, Department of Psychiatry and Psychotherapy, Medical Center – University of Freiburg, Faculty of Medicine, University of Freiburg, Hauptstraße 5, D-79104 Freiburg, Germany; 2 Institute of Human Genetics, Medical Center – University of Freiburg, Faculty of Medicine, University of Freiburg, Breisacher Straße 33, D-79106 Freiburg, Germany

**Keywords:** Autism spectrum disorder (ASD), Tourette syndrome, *SCN2A*, *SCN3A*

## Abstract

**Background:**

Mutations in voltage-gated sodium channel (*SCN*) genes are supposed to be of importance in the etiology of psychiatric and neurological diseases, in particular in the etiology of seizures. Previous studies report a potential susceptibility region at the chromosomal locus 2q including *SCN1A*, *SCN2A* and *SCN3A* genes for autism spectrum disorder (ASD). To date, there is no previous description of a patient with comorbid ASD and Tourette syndrome showing a deletion containing *SCN2A* and *SCN3A*.

**Case presentation:**

We present the unique complex case of a 28-year-old male patient suffering from developmental retardation and exhibiting a range of behavioral traits since birth. He received the diagnoses of ASD (in early childhood) and of Tourette syndrome (in adulthood) according to ICD-10 and DSM-5 criteria. Investigations of underlying genetic factors yielded a heterozygous microdeletion of approximately 719 kb at 2q24.3 leading to a deletion encompassing the five genes *SCN2A* (exon 1 to intron 14–15), *SCN3A, GRB14* (exon 1 to intron 2–3), *COBLL1* and *SCL38A11.*

**Conclusions:**

We discuss the association of *SCN2A, SCN3A, GRB14, COBLL1* and *SCL38A11* deletions with ASD and Tourette syndrome and possible implications for treatment.

## Background

Mutations in neuronal voltage gated sodium channel genes such as *SCN1A*, *SCN2A* and *SCN3A* are supposed to play an important role in psychiatric disorders and neurological diseases [1]. These three sodium channel genes encoding distinct α-subunit isoforms are clustered within 600 kB on chromosome 2q24 and are highly expressed in neurons and glia throughout the central and peripheral nervous system [2, 3].

Genome-wide scan studies for autism susceptibility genes reported a potential susceptibility region at locus 2q including *SCN1A*, *SCN2A* and *SCN3A* genes [4, 5].

Dravet-Syndrome (DS) is a childhood disorder associated with mainly loss-of-function mutations in the *SCN1A* gene. It is characterized by frequent seizures and devastating effects on cognitive and behavioural development, persisting into adulthood [6]. While approximately 70–80% of DS patients show mutations in *SCN1A*, some patients have variants in other genes including *SCN2A* [7].

Mutations of the *SCN2A* gene have also been reported in patients with autism [8] as well as with benign familial infantile seizures, intractable epilepsy, infantile spasms and severe myoclonic epilepsy of infancy [9–12].

Previous investigations mainly led to case reports with deletions on chromosome 2q24 being associated with seizures complicated by neurobehavioral comorbidities such as cognitive impairment, psychiatric disorders and social problems [13–15]. To date, there are only two published cases with deletions on chromosome 2q24 displaying a phenotype with autistic features and developmental delay, but no seizures: Celle et al. [2] described the case of a 3-year old boy presenting with autistic features, language delay, microcephaly and no history of seizures. He had an interstitial deletion of ~ 291.9 kb at band 2q24.3 with loss of the entire *SCN2A* gene and part of the *SCN3A* gene. Another case report described a 3 years and 4 months old girl with autistic features, developmental delay, mental retardation, language impairment and dysmorphic features, who carried a 2.8 Mb de novo deletion on chromosome 2q24.2 containing nine genes including *SCN2A2*, *SCN3A*, *GRB14* and *COBLL1* as well as *LOC643397*, *FLJ39822*, *LOC643405*, *FIGN* and *TAIP-2* [16]. Until now, there is no description of a patient with *SCN* gene mutations and autism spectrum disorder (ASD) with additional symptoms of Tourette syndrome.

## Case presentation

We present the unique case of a 28-year-old male patient displaying a complex clinical picture with mental retardation and various behavioural problems since birth. Symptoms of the autism spectrum comprising difficulties in social interaction and communication are reported since childhood. Additionally, he suffers from auto-aggressive tics in terms of beating himself with objects against his head and lower jaw, head movement tics and simple vocal tics. Striking dysmorphic features are not evident.

### Developmental and somatic history

Except a one-time bleeding in week 20 of gestation, pregnancy had been without any complications. No infections, medication, smoking, or intake of alcohol or drugs during pregnancy was reported. The patient was delivered in week 40 of gestation with the help of a ventouse due to irregular cardiac activity. During delivery there were minor signs of birth asphyxia. Birth weight was 2.900 g (25th percentile), birth length 51 cm (50th percentile) and head circumference 33 cm (<3rd percentile). During infancy a prominent frontal fissure was conspicuous. A premature ossification of the sagittal fissure could not be detected.

The patient showed psychomotor retardation: he walked alone only by 26 months and was not able to sit alone falling over to one side without shoring up even at the age of 2. Furthermore, tics in terms of eye blinking as well as a muscular hypotonia were described. The patient’s parents reported early autistic features such as difficulties in social communication and interaction with avoiding eye contact and poor interest in social interaction. Development of speech was delayed (first words with 18 months). He refused body contact and demonstrated stereotypic patterns of behaviour such as filling bowls without showing any variations.

When examined at the age of 27 months, the patient presented some special facial features such as synophrys, epicanthus, modelled ears, a deep joined thumb and microcephaly. His weight was 10 kg (3rd percentile), his length 88 cm (25th percentile) and his head circumference 46 cm (2 cm above the 3rd percentile). The patient only spoke a few words and never a whole sentence. Mostly, he only repeated the words he had heard before in terms of an echolalia. A considerable general delay of development with severe perceptual disturbance and autistic traits was diagnosed.

Since the age of two years, the patient shows relevant aggressive symptoms such as throwing his head on the floor or biting into items. He needs extensive support concerning all activities of daily living and requires constant daily routines. During nursery school, auto-aggressive symptoms exacerbated in terms of head banging behaviour injuring his jaw and ears. At the age of 3 years, he started grinding his teeth. Later on, he presented head throwing movements against his left shoulder and banging of one row of his teeth against the other. There was a pattern of aggravation of the tic symptoms during stressful situations. He repeatedly showed refusal of meals and sleeping disturbances. At the age of 3 years, once there was a query febrile convulsion associated with an infection. Apart from that, there was no evidence for further seizures and clearly no epilepsy.

According to ICD-10 and DSM-5 classification, the clinical features of the patient are consistent with early infantile autism as well as with Tourette syndrome.

In the clinical examination, the patient presented with cauliflower-ears as a result of his head banging behaviour and subsequent repetitive ear injuries as well as various injuries of all kinds and healing stages. Dysmorphic features or additional external malformations were not noted, there was no evidence of internal abnormalities (heart, eye, inner ear) either. The patient showed a preserved ability of speech comprehension with rare speech production.

### Family history

Our patient is the first son of non-consanguineous healthy German parents. His younger brother (24 years old) was diagnosed with an autism spectrum disorder (Asperger syndrome). There was no evidence of syndromal-secondary autism in the brother. He did not show intellectual impairment, dysmorphic signs or organic conditions such as a heart malformation or epilepsy. The mother’s paternal grandparents were cousins. They had five healthy children. In their further progeny three mentally retarded persons were reported, one granddaughter (died at the age of approximately 27 years) and two great-grandchildren (a boy and a girl). The exact diagnosis was unknown to our patient’s parents. Consanguinity of the retarded relatives’ parents was denied. One of the patient’s cousins (the son of his father’s brother) was said to suffer from clinically apparent dyslexia and perhaps an autism spectrum disorder. A disablement of a cousin of our patient’s father (son of his father’s sister) could not be specified further. An assessment of the father revealed no clinical characteristics, neither with respect to striking dysmorphic features, the occurrence of tics nor with respect to autistic symptoms. Further relatives with neurodevelopmental disorders were not reported on the father’s side of the family.

### Cytogenetic and array-analysis

Conventional R-banded karyotyping of the patient was performed according to standard protocols with a resolution of approximately 500 bphs and revealed a structurally and numerically normal male karyotype (46,XY) in all 21 metaphases examined.

**Fig. 1:**
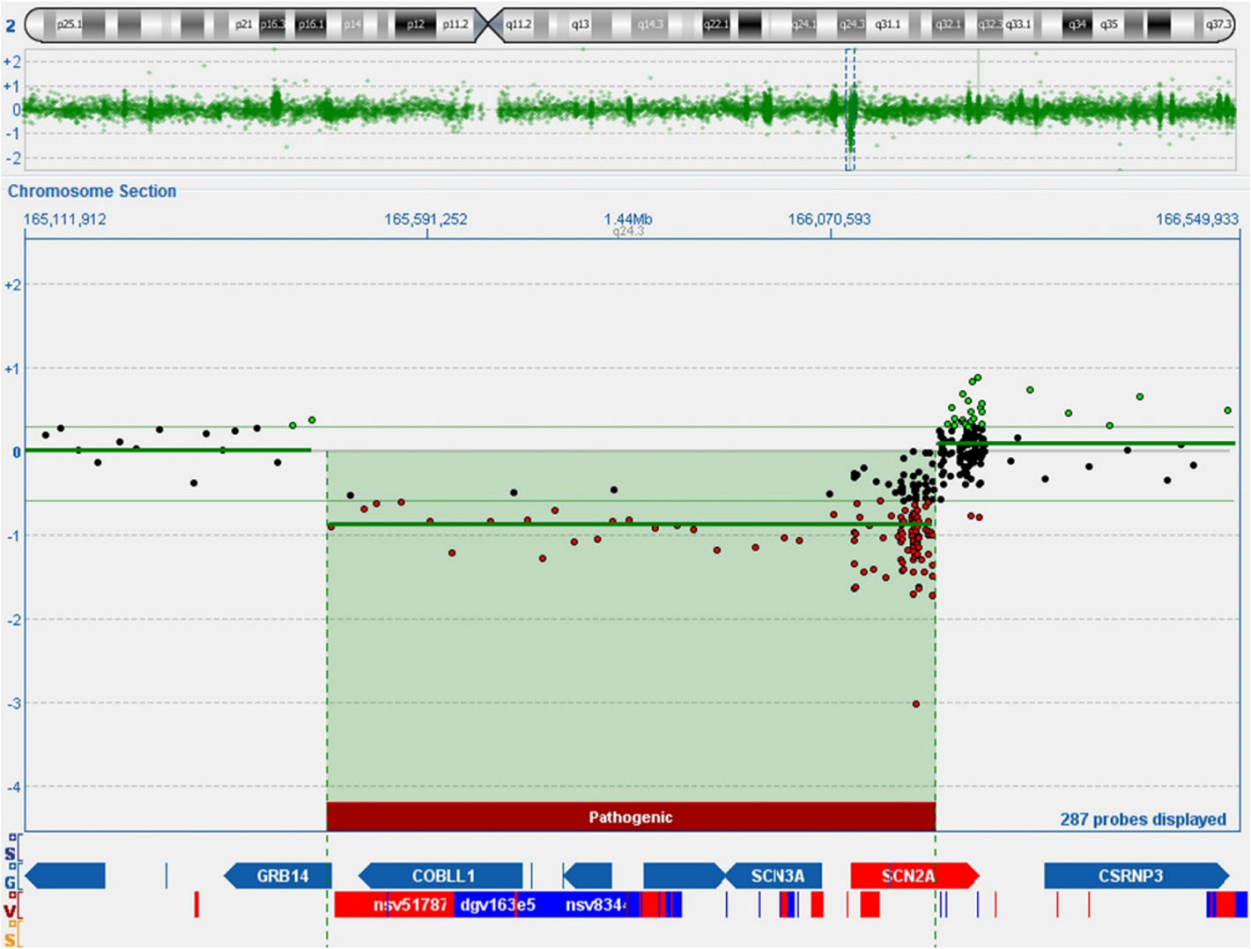
Results of microarray-analysis. Molecular karyotyping revealed a deletion of approximately 719 kb out of the chromosomal region 2q24.3 encompassing the 5 genes *GRB14* (exon 1 to intron 2–3), *COBLL1*, *SLC38A11*, *SCN3A* and *SCN2A* (exon 1 to intron 14–15) listed in OMIM (Online Mendelian Inheritance in Man)

Furthermore, the genomic DNA of the patient was examined by microarray-analysis (CytoSureTM Constitutional v3 Array 180 k, OGT (Oxford Gene Technology)) according to the manufacturer’s instructions. After hybridization, the array was scanned with the SureScan Microarray scanner (Agilent), the results were analyzed using CytoSure interpret software v.4.9 (OGT) against the Genome Reference Consortium human genome GRCh37 (hg19). Molecular karyotyping revealed a heterozygous deletion of approximately 719 kb (267 contiguous oligonucleotides) out of the chromosomal region 2q24.3 (karyotype according to ISCN (International System for Human Cytogenetic Nomenclature) (2016): arr[GRCh37] 2q24.3(165471418_166190427)× 1). The deletion encompasses the five genes *GRB14* (exon 1 to intron 2–3), *COBLL1*, *SLC38A11*, *SCN3A* and *SCN2A* (exon 1 to intron 14–15) listed in OMIM (Online Mendelian Inheritance in Man) (Fig. 1). With FISH (fluorescence in situ hybridization) analysis using probe RP11-150F4 (Empire Genomics) located at 2q24.3 the deletion could be confirmed. There is no evidence in literature that the microdeletion we detected is a common variant in the Caucasian population [17, 18]. “Orphanet” states a prevalence rate of a 2q24 microdeletion < 1/1000000 (worldwide) (https://www.orpha.net/data/patho/GB/uk-2q24.pdf).

The subsequently performed chromosome analysis of the parents, FISH with RP11-150F4 included, showed a normal female karyotype in the mother and a normal male karyotype in the father with no evidence of a deletion or rearrangement at 2q24.3. Therefore, it can be asserted that the deletion in the patient is a de novo mutation. The patient’s younger brother (suffering from Asperger syndrome) was shown to have a normal male karyotype without a deletion or rearrangement at 2q24.3.

### Other investigations

Magnetic resonance imaging conducted in 2014 showed no abnormalities of the patient’s brain. In 1991, a detailed investigation was performed with a metabolic screening of serum, urine and cerebrospinal fluid revealing normal results. In the EEG, there was a slow baseline activity without any epilepsy suspicious potentials. A proton-spectroscopy showed no abnormalities of N-acetyl-aspartate, choline and phosphor-creatinine levels. An ophthalmologic investigation exhibited no deviations. In 1997, gastrointestinal passage was unsuspicious, and in an X-ray of the brain no stenosis of the sagittal fissure was confirmed.

## Discussion and conclusions

In the present case report, we describe a 28-year-old male patient with symptoms of an early infantile autism as well as a Tourette syndrome. He carries a 719 kb deletion on chromosome 2q24.3 including the genes *SCN3A* and *SCN2A* which code for voltage-gated sodium channels. To date, no similar case has been described in the literature with respect to the phenotype of autism spectrum disorder plus Tourette syndrome but without epilepsy. The patient showed a global retardation of development (including psychomotor and language delay), various autistic traits, echolalia, severe auto-aggressive symptoms in terms of head banging, tic-specific head movements and vocal tics. There might have been one febrile convulsion at age 3; however, clearly there was no diagnosis of epilepsy. No other seizures had ever been reported. The somatic abnormalities included dysmorphic features such as synophrys, epicanthus, modelled ears, a deep joined thumb and microcephaly.

To date, there are only two case reports referring to patients with autistic features, language impairment, dysmorphic features and no history of seizures presenting a deletion of *SCN2A* and *SCN3A* genes [2, 16]. Chen et al. [16] described the most similar case to the one presented in this report. He presented a 3 years and 4 months old girl with autistic features, developmental delay, mental retardation, language impairment, dysmorphic features and no history of seizures with a 2.8 Mb de novo deletion on chromosome 2q24.2 entailing loss of nine genes including *SCN2A2*, *SCN3A*, *GRB14* and *COBLL1.*

Bartnik et al. [13] identified a de novo ~ 110 kb deletion involving exons 1–2 of *SCN2A* and non-coding exon 1a of *SCN3A* in a 25-year-old female with mental retardation, neurobehavioural and psychiatric abnormalities and a history of infantile seizures. Additionally, there are several other case reports presenting patients with a deletion on chromosome 2q24 with current seizures or a history of seizures [13–15]. In all previous case reports, no additional symptoms of Tourette syndrome have been described to date.

The presently available literature suggests that mutations in *SCN2A* can be causative for ASD, benign familial neonatal infantile seizures, intractable epilepsy, infantile spasms and severe myoclonic epilepsy of infancy [8–12].

Since sodium channels are critical for action potential generation and propagation, a causative association between seizures and sodium channel dysfunction is plausible, whereas it is more difficult to understand this link for ASD and tics [1]. It is proposed that loss of function *SCN1A* mutations associated with seizures cause reduced GABA release leading to an inappropriate inhibition in related neuronal networks [6]. Parvalbumin neurons represent inhibitory GABAergic cells that are involved in various forms of feed-forward inhibition within the striatum [19]. Postmortem studies in patients with Tourette syndrome demonstrate a consistent and profound imbalance of parvalbumin-positive neuronal distribution in the basal ganglia [20]. The selective deficit of parvalbumin-positive and cholinergic striatal interneurons in Tourette syndrome was supposed to result in an impaired cortico/thalamic control of striatal neuron firing [21]. Evidence from studies demonstrates that Na(v)1.2 (the protein encoded by *SCN2A*) is abundant in parvalbumin-positive GABAergic inhibitory interneurons, at least in the hippocampus and the temporal lobe [22].

Apart from *SCN2A* and *SCN3A* genes being affected by the presently detected deletion, *GRB14* (exon 1 to intron 2–3), *COBLL1* and *SLC38A11* were also deleted in our patient. Thus, heterozygous deletion of these genes might also play a pivotal role in conferring ASD symptoms and could even be suggested to confer symptoms of Tourette syndrome in the present case: Loss of *SLC38A11*, a putative sodium-coupled neutral amino acid transporter [23] might enhance the effects of *SCN2A* and *SCN3A* deletion. The *COBLL1* gene, encoding the Cordon-Bleu WH2 Repeat Protein Like 1 was suggested to be a negative regulator of apoptosis and associated with lower insulin resistance [24]. A deletion on chromosome 2q24.4 encompassing 47 genes including *SCN2A2*, *SCN3A* and *COBLL1* was reported in a patient with severe epilepsy [14]. *GRB14* encodes a growth factor receptor-binding protein acting as an inhibitor of intracellular signaling pathways regulating growth and metabolism [25]. One previous investigation reported a deletion on chromosome 2q comprising *GRB14* and *COBLL1* in a patient with autistic features, developmental delay, mental retardation, language impairment and dysmorphic features. In this case, additional repetitive hand movements have been described [2, 16]. Therefore, it is possible that deletions of *GRB14* and *COBLL1* may contribute to tic symptoms. An association of the above mentioned affected genes with Tourette syndrome proper, however, has not been reported in the presently available literature. From our point of view, the deletion therefore adequately explains the patient’s symptomatology. Furthermore, whole genome sequencing could provide further information on whether additional mutations/variants are present in the patient’s autistic brother contributing to the development of autistic symptoms in the patient.

Concerning treatment of *SCN* gene mutations, existing literature is really scarce. A causal treatment of deletions in chromosomal area 2q24.3 is not available. To date, a substitution of the missing gene products of the patient is not possible.

Promising studies reported a *SCN2A* mutation in a Chinese boy with infantile spasm responding to a modified Atkins Diet [26]. It is an “alternative” ketogenic diet with results demonstrating efficacy in the treatment of intractable seizures [27]. Evidence shows that ketones produced after a modified Atkins Diet could reduce neuron excitability by inhibiting glutamate transport and activating ATP-sensitive potassium channels [28]. Currently, there are no publications available investigating the effect of a ketogenic diet on Tic disorders or Tourette syndrome.

A review concluded that for the treatment of DS, which is often caused by loss-of-function mutations in *SCN1A*, valproate and benzodiazepines should be the first-line treatment but are often insufficient. Topiramate and levetiracetam, bromide and ketogenic diet provide efficacy as adjunctive therapy. Lamotrigine and carbamazepine should be avoided as they are supposed to lead to an increased occurrence of seizures [29]. Another promising drug is stiripentol showing efficacy in a combination with valproate and clobazam [29, 30]. Among patients with DS, a treatment with cannabidiol resulted in a reduction in seizure frequency compared to placebo, nevertheless higher rates of adverse events have been reported [31].

For the future, further research into targeted treatment options for *SCN2A* and *SCN3A* deletions is warranted. Previous studies primarily focused on patients with seizures and reported about medication with antiepileptic drugs or Atkins diets. In our case, the patient presented additional symptoms of Tourette syndrome, but no epilepsy. It remains to be elucidated whether the heterozygous loss of *SCN2A* and *SCN3A* or *GRB14*, *COBLL1* and *SLC38A11*, respectively, might also contribute to the development of tics, which remains subject to investigation in large hypothesis-driven association studies.

## Abbreviations

ASD: autism spectrum disorder
ATP: adenosine triphosphate
COBL: Cordon-Bleu
DS: Dravet-Syndrome
DSM: Diagnostic and Statistical Manual of Mental Disorders
EEG: electroencephalography
FISH: fluorescence in situ hybridization
GABA: gamma-aminobutyric acid
GRB: growth factor receptor-bound protein
ICD: International Statistical Classification of Diseases
ISCN: International System for Human Cytogenetic Nomenclature
OGT: Oxford Gene Technology
OMIM: Online Mendelian Inheritance in Man
SCN: sodium voltage-gated channel
SLC: solute carrier
